# Mercurius-vivus

**Published:** 1896-05

**Authors:** 


### HOMŒOPATHIC PHYSICIAN,

A MONTHLY JOURNAL OF

HOMŒOPATHIC MATERIA MEDICA AND CLINICAL MEDICINE.

"If our school ever gives up the strict inductive method of Hahnemann, we are lost, and deserve only to be mentioned as a caricature in the history of medicine."—CONSTANTINE HERING.

Vol. XVI. MAY, 1896. No. 5.

### EDITORIAL.

MERCURIUS-VIVUS.—In the last number were given some of the mental symptoms and a few of the head symptoms of Mercury, together with some valuable notes upon the sense of a band around the head.

Attention is now drawn to the symptom of Mercurius—"Headache, as if the head would burst, with fullness of the brain." This is similar to Natrum-muriaticum. Belladonna has this bursting headache. It is thus expressed by Lippe in his *Materia Medica:* "Pressing headache, as if the head would split." In Hering's *Condensed Materia Medica* it is expressed as follows: "Frequently obliged to stand still in walking, from the violence of the pain in the forehead. At every step it seemed as if the brain rose and fell in the forehead." Again: "Pain, as if the bones were being lifted up."

Cannabis-indica has pain as if top of the head were opening and shutting and as if the calvarium were being lifted up.

China, headache so severe it seems as if the skull would burst.

On this subject of bursting headache a formidable list of remedies having this symptom can be found in the second chapter of Dr. Lee's *Repertory*, under the head of "Bursting," at page 86. Another equally formidable list of remedies having this symptom, together with all the details by which they may be differentiated from one another, may be found in Dr. Knerr's great *Repertory*, at page 122.

Mercurius has dry, stinging, burning, fetid eruption, like yellow crusts, on the forepart of the head and temples; when scratching, inflammation and erysipelas. This symptom, Dr. Lippe stated, was a great characteristic, indicating Mercurius. Mercurius has open fontanelles. This is similar to Calcarea-carbonica and Silicea.

Mercurius has fœtid, sour-smelling, oily perspiration on the forehead, which is icy-cold. Fœtid perspiration is characteristic of Silicea. Sour perspiration indicates Calcarea. Cold perspiration on cold forehead indicates Carbo-vegetabilis and Veratrum. Oily perspiration is the keynote of Mercury.

Cold perspiration, standing in beads on the forehead, is the keynote of Veratrum. This symptom occurring in cases of cholera morbus, cholera infantum, whooping-cough, vomiting, and other affections, almost certainly indicates Veratrum, and will lead to a brilliant cure. The editor can give abundant testimony to the value of this symptom in curing quickly, almost magically, cholera infantum, persistent vomiting, whooping-cough and the ordinary cough of a "bad cold."

There is a young woman who sometimes comes into this office for treatment, who, when a year and a half old, was given up to die from cholera infantum, by her allopathic attendant. The writer of this editorial was then called in and cured her with Veratrum, being led to think of it by this symptom of cold perspiration on the forehead.

Tartar-emetic has cold perspiration on the forehead in cases of croup, along with its well-known Guernsey keynote "rattling in the larynx as if a cupful of mucus were lodged there, yet none is expectorated."

The editor can testify to the efficacy of this keynote also, having had repeated success with it in cases of croup.

He cured croup three times in the same child, occurring once every winter for three years in succession. The indication for Tartar-emetic was always "loud rattling of mucus in the larynx, both in breathing and coughing, yet none is expectorated or dislodged." There was also cold sweat upon the forehead. On each occasion it was apparent that the child had but few hours to live. The administration of Tartar-emetic in the two hundreth potency, without assistance from any kind of "adjuvant" treatment, quickly relieved the child and later cured it. The child is now grown up and is the mother of children herself.

Cold perspiration on forehead occurs under other remedies. It may be studied by consulting Dr. Knerr's *Repertory*, page 1115. What is here given, however, is simply from Dr. Lippe's lectures, and, therefore, exhaustive comparisons of a large number of remedies would be out of place and unnecessary, as they can be found in the new *Repertory to the Guiding Symptoms*.

